# Integrating Whole-Exome Sequencing with Structural Neuroimaging in Bipolar Disorder

**DOI:** 10.1192/j.eurpsy.2026.10707

**Published:** 2026-07-31

**Authors:** K.-M. Han, M.-R. Han, B.-J. Ham

**Affiliations:** 1 Department of Psychiatry, Korea University College of Medicine, Seoul; 2 Division of Life Sciences, Incheon National University College of Life Sciences and Bioengineering, Incheon, Korea, Republic Of

## Abstract

**Introduction:**

Bipolar disorder (BD) is a highly heritable psychiatric condition, with heritability estimated at ~60%–85%. Advances in neuroimaging and imaging-genetic methods enable characterization of genetic influences on neural phenotypes, emotion, behavior, and cognition in BD. Integrating whole-exome sequencing (WES) with neuroimaging may clarify heritable neural correlates of BD and the variants that contribute to them.

**Objectives:**

To perform WES in patients with BD and healthy controls (HC) to evaluate gene-level rare-variant burden and to test the effects of single-nucleotide polymorphisms (SNPs) on cortical thickness and white-matter tract integrity.

**Methods:**

WES was conducted in 93 patients with BD and 161 HCs to identify germline variants, copy-number alterations (CNAs), and gene-based rare-variant effects on BD. A neuroimaging subset (82 BD, 154 HC) underwent T1-weighted MRI and diffusion tensor imaging (DTI). Cortical thickness was estimated with FreeSurfer using the Destrieux parcellation, and DTI metrics from 18 major tracts were derived with TRACULA. Imaging–genetic association analyses evaluated SNP and rare-variant effects on cortical thickness and tract integrity.

**Results:**

In patients with BD, rare coding variants were observed across 382 genes; *SYNE1*, *PKHD1L1*, *FRAS1*, and *ZFHX4* were most frequently affected (>20% with non-silent variants). Gene-based testing implicated *GNB3*, with a higher burden of rare nonsynonymous variants associated with BD status. Recurrent focal CNAs at 5q13.2 encompassing *BDP1* were also detected. Integrating WES and neuroimaging, homozygosity for the *KMT2C* rs56850341 risk allele (GG) was associated with widespread reductions in white-matter integrity and thinner cortex in the right lingual gyrus in the full sample.

**Conclusions:**

Rare nonsynonymous variants in *GNB3* may contribute to BD liability, and variation in *KMT2C* may influence brain structural phenotypes. These findings link genetic variation to structural neural alterations in BD and motivate replication in larger, independent cohorts.

**Disclosure of Interest:**

None Declared

